# Interpretable Machine Learning for Serum-Based Metabolomics in Breast Cancer Diagnostics: Insights from Multi-Objective Feature Selection-Driven LightGBM-SHAP Models

**DOI:** 10.3390/medicina61061112

**Published:** 2025-06-19

**Authors:** Emek Guldogan, Fatma Hilal Yagin, Hasan Ucuzal, Sarah A. Alzakari, Amel Ali Alhussan, Luca Paolo Ardigò

**Affiliations:** 1 Department of Biostatistics, and Medical Informatics, Faculty of Medicine, Inonu University, 44280 Malatya, Turkey; emek.guldogan@inonu.edu.tr (E.G.); hasan.ucuzal@inonu.edu.tr (H.U.); 2 Department of Biostatistics, Faculty of Medicine, Malatya Turgut Ozal University, 44210 Malatya, Turkey; 3 Department of Computer Sciences, College of Computer and Information Sciences, Princess Nourah bint Abdulrahman University, P.O. Box 84428, Riyadh 11671, Saudi Arabia; 4 Department of Teacher Education, NLA University College, Linstows Gate 3, 0166 Oslo, Norway

**Keywords:** breast cancer, metabolomics, explainable AI, LightGBM, SHAP, biomarkers, diagnostic accuracy

## Abstract

*Background and Objectives:* Breast cancer accounts for 12.5% of all new cancer cases in women worldwide. Early detection significantly improves survival rates, but traditional biomarkers like CA 15-3 and HER2 lack sensitivity and specificity, particularly for early-stage disease. Advances in metabolomics and machine learning, particularly explainable artificial intelligence (XAI), offer new opportunities for identifying robust biomarkers and improving diagnostic accuracy. This study aimed to identify and validate serum-based metabolic biomarkers for breast cancer using advanced metabolomic profiling techniques and a Light Gradient Boosting Machine (LightGBM) model. Additionally, SHapley Additive exPlanations (SHAP) were applied to enhance model interpretability and biological insight. *Materials and Methods:* The study included 103 breast cancer patients and 31 healthy controls. Serum samples underwent liquid and gas chromatography–time-of-flight mass spectrometry (LC-TOFMS and GC-TOFMS). Mutual Information (MI), Sparse Partial Least Squares (sPLS), Boruta, and Multi-Objective Feature Selection (MOFS) approaches were applied to the data for biomarker discovery. LightGBM, AdaBoost, and Random Forest were employed for classification and to identify class imbalance with the Synthetic Minority Oversampling Technique (SMOTE). SHAP analysis ranked metabolites based on their contribution to model predictions. *Results:* Compared to other feature selection approaches, the MOFS approach was more robust in terms of predictive performance, and metabolites identified by this method were used in subsequent analyses for biomarker discovery. LightGBM outperformed the AdaBoost and Random Forest models, achieving 86.6% accuracy, 89.1% sensitivity, 84.2% specificity, and an F1-score of 87.0%. SHAP analysis identified 2-Aminobutyric acid, choline, and coproporphyrin as the most influential metabolites, with dysregulation of these markers associated with breast cancer risk. *Conclusions:* This study is among the first to integrate SHAP explainability with metabolomic profiling, bridging computational predictions and biological insights for improved clinical adoption. This study demonstrates the effectiveness of combining metabolomics with XAI-driven machine learning for breast cancer diagnostics. The identified biomarkers not only improve diagnostic accuracy but also reveal critical metabolic dysregulations associated with disease progression.

## 1. Introduction

Breast cancer is a major global health burden and is responsible for 12.5% of all new cases of cancer in women per year [1]. This malignancy has a considerable impact on public health due to its prevalence as a leading malignancy in women. While the incidence of breast cancer varies geographically, its incidence rates tend to be higher in developed areas of North America and Europe, but increasing rates in developing areas of Asia and Africa are a cause for concern. The phenomenon of rising incidence rates is mainly due to changes in ways of living, more urbanization, and associated risk factors like changed nutritional patterns, dips in physical activity, and a rise in exposure to environmental pollutants [2]. About 2.3 million new breast cancer cases were diagnosed worldwide in 2020, which highlights the need for prevention and treatment, according to experts. However, during early detection, treatment outcomes and, ultimately, patient survival rates are improved. The five-year survival rate for localized breast carcinomas is 90 percent or better. In contrast, advanced-stage breast cancers are associated with poor prognosis, with five-year survival rates typically below 30%. This enormous differences emphasize the important role of early diagnosis and early intervention in the maximization of treatment efficacy and patient benefits [2]. While traditional biomarkers such as CA 15-3 and HER2 are widely used, their limited sensitivity, especially in early-stage breast cancer, underscores the need for innovative diagnostic approaches. Thus, it is underscored that novel biomarkers for improving early detection and patient outcomes are needed. Gradient boosting algorithms, such as LightGBM, have recently demonstrated potential for analyzing relatively complex metabolomic data for improved diagnostic accuracy and interpretability in cancer research [3].

Recently, metabolomics has become a revolutionary method to understand cancer biology. The systematic identification and quantification of small molecules present in biological samples is the focus of this field, and these individual molecules provide insights into altered metabolic pathways in disease states. Metabolomics directly represents biochemical activity and cellular processes, which makes it a particularly attractive approach to biomarker discovery [4]. In breast cancer, metabolomics research has indicated significant changes in lipid metabolism; amino acid pathways and energy production processes have been observed, and it has been found that they can be used as potential diagnostic and prognostic markers. Low-molecular-weight serum-based metabolic biomarkers, including aspartate, glycerol-phosphate, and lipid metabolites, have been demonstrated to reliably distinguish breast cancer patients from healthy controls. Levels of glycerol-phosphate, certain lipids, and a reduction in circulating aspartate are correlated with tumor progression. Published studies show that combining multiple serum biomarkers increases diagnostic accuracy, and some achieve sensitivity and specificity of over 90 percent in the detection of early-stage breast cancer [1]. While these advances have been achieved, reproducibility remains somewhat elusive because of the differences in analytical methods, population diversity, and study design [5]. Further standardization of these inconsistencies through standardized methodologies, more robust validation protocols, and multi center studies could further improve the value of metabolomics in breast cancer biomarker discovery [6].

Advanced metabolomic profiling techniques are used in this study to identify and validate serum-based metabolic cancer biomarkers for breast cancer. The researchers produce integrated explainable artificial intelligence (XAI) models by employing liquid chromatography–time-of-flight mass spectrometry (LC-TOFMS) and gas chromatography–time-of-flight mass spectrometry (GC-TOFMS) coupled with robust statistical analyses to improve the interpretability of the results. Previous studies using AI have investigated serum metabolomic profiling in breast cancer without integrating and applying XAI techniques to AI and machine learning approaches [7,8,9], highlighting the novelty of integrating SHAP-based interpretability in our study.

Therefore, the XAI-integrated prediction models used in this study will enable clinicians to understand the specific metabolic pathways underlying breast cancer through the contributions of individual metabolites. The aim of this study is not only to find novel metabolites and pathways that distinguish breast cancer patients from healthy controls, but also to determine metabolite selectivity over other malignancies. XAI enables interpretability and transparency of the decision-making process behind biomarker identification to address key gaps in the literature and clinical practice.

## 2. Materials and Methods

### 2.1. Study Sample and Power Analysis

Existing open access metabolomics panel data obtained from serum samples of 103 breast cancer patients and 31 healthy controls were used in the study. Given the substantial mean difference in 2-Aminobutyricacid levels between the groups, a post hoc power analysis was conducted. Using the observed effect size (Cohen’s d = 4.14, calculated from the mean difference and pooled standard deviation), the relevant analysis revealed exceptional statistical power (>99%) at α = 0.001. This outcome indicates a high probability of reliably detecting such a large intergroup difference in future studies with comparable designs [10].

### 2.2. Sample Collection and Storage

All participants provided fasting blood samples in the morning to minimize diurnal variations in metabolite levels, following standardized collection protocols. The samples were processed according to rigorous pre-analytical procedures. Serum samples were separated by centrifugation at 3000 rpm for 10 min and subsequently stored at −80 °C until further analysis. The cryogenic storage conditions were continuously monitored to maintain sample stability. Across all cohorts, the collection protocols ensured consistency in terms of sample quality and comparability. Throughout the study, the sample collection, processing, and storage procedures were strictly standardized to minimize pre-analytical variables. This approach was critical for ensuring the reliability and reproducibility of the resulting data. All samples were collected under standardized fasting conditions and with rigid sample preparation and storage protocols, as well as stringent protocols for the continuous monitoring of cryogenic conditions, to maintain metabolite stability [10].

### 2.3. Metabolomics Profiling

LC-TOFMS and GC-TOFMS technologies were used in a comprehensive metabolomic analysis using a dual-platform approach. An optimized two-step extraction procedure was used for sample preparation to maximize metabolite recovery while minimizing possible matrix effects that could degrade analysis. The analytical instrumentation included a gas chromatograph (GC) with an Agilent 7890 (Agilent, Santa Clara, CA, USA) interfaced to a LECO Pegasus HT mass spectrometer (LECO Corporation, St. Joseph, MI, USA) for GC-TOFMS analysis, and an ACQUITY UPLC (Waters, Milford, MA, USA) coupled to a SYNAPT G2 (Waters, Milford, MA, USA) for LC-TOFMS analysis. For the careful control of separation conditions, chromatographic separations and mass spectrometric analyses were performed. Analytical reliability and reproducibility were ensured by rigorously validated data acquisition and processing that adhered to rigorously validated protocols, utilizing quality control samples and internal standards. Using this methodological approach, the complete metabolome was profiled with high analytical precision [4].

### 2.4. Data Analyses

Statistical analyses were performed to compare metabolite levels between the groups. The normality of data distribution was assessed using the Shapiro–Wilk test. For metabolites with non-normal distributions, the Mann–Whitney U test was applied, while the independent samples *t*-test was used for normally distributed variables. All tests were two-tailed, and statistical significance was set at *p* < 0.001. Analyses were conducted using SPSS Statistics v28 (IBM Corp., Armonk, NY, USA).

### 2.5. Machine Learning Pipeline

In this study, four different feature selection methods were evaluated in the first stage to determine the biomarker candidate serum metabolomics to be used in the classification model to distinguish breast cancer patients and healthy individuals: Mutual Information (MI), Sparse Partial Least Squares (sPLS), Boruta, and Multi-Objective Feature Selection (MOFS). Biomarker candidate metabolites were selected by calculating the information gain between metabolites and outputs with the MI method. MI is an information theory-based method that measures the statistical dependence between two random variables. The MI value between the target variable (Y) and an attribute (X) is calculated by the formula I(X;Y) = H(Y) − H(Y∣X), where H(Y) is the entropy and H(Y∣X) is the conditional entropy. Attributes with high MI values are considered more informative in predicting the target variable. MI is especially effective in complex data structures because it can capture nonlinear relationships [11,12,13,14]. sPLS is a dimensionality reduction and variable selection method that combines principal component analysis (PCA) and least squares regression. The model creates latent variables that maximize covariance with the target variables while adding an L1 penalty term to ensure sparsity. In this way, only the most important attributes have non-zero weight coefficients. sPLS provides stability in high-dimensional data (n < p) and can be applied to both regression and classification problems. The sPLS approach determined significant metabolites based on the average of the absolute values of PLSRegression coefficients [15,16,17]. Boruta is a Random Forest-based wrapper method based on the concept of “shadow features”. Shadow features, created from random permutations of original features, are compared with the real features to test statistical significance. An attribute is considered “important” if its average importance score exceeds the maximum score of shadow features. Boruta enables robust selection by aggressively eliminating unnecessary variables, and prevents overfitting with multiple testing corrections [18]. The MOFS [19] approach provided a multi-objective and stepwise selection strategy that included metabolites that were selected jointly by at least two methods among the MI, sPLS, and Boruta methods. After employing the optimal feature selection method, models were built with various classification algorithms, including LightGBM, AdaBoost, and Random Forest, and model performance was evaluated by calculating the accuracy, sensitivity, specificity, F1-Score, and AUC metrics by performing 10-fold cross-validation with the selected biomarker candidate metabolomics dataset. The machine learning pipeline was constructed using LightGBM, a high-performance gradient boosting framework optimized for efficiency and accuracy in large-scale datasets [20]. For comparative analysis, the AdaBoost and Random Forest models were also implemented to evaluate performance across diverse architectures. Performance metrics were prioritized to address the clinical imperative of minimizing false negatives (e.g., missed cancer diagnoses) and false positives (e.g., unnecessary invasive procedures) in oncology. These assessment metrics were prioritized to address the clinical need for minimizing false negatives (high sensitivity) and false positives (high specificity) in cancer diagnostics. All models were developed in Python (v3.8) using scikit-learn and pandas, leveraging their reproducibility advantages and extensive library support.

### 2.6. Hyperparameter Optimization

Hyperparameters were systematically optimized via Bayesian optimization with the Tree-structured Parzen Estimator (TPE) algorithm [21]:

- LightGBM: Learning rate = 0.05; num_leaves = 31; max_depth = 7; n_estimators = 500.
- AdaBoost: Base estimator = Decision Stump; n_estimators = 100; learning rate = 0.8.
- Random Forest: n_estimators = 300; max_depth = 10; max_features = “sqrt”.

For hyperparameter optimization, we employed a budget of 50 iterations (trials) per model to sufficiently explore the hyperparameter space. The TPE algorithm efficiently explored high-dimensional parameter spaces using probabilistic surrogate models, outperforming grid/random search. The TPE algorithm was selected over grid or random search for its ability to efficiently explore high-dimensional parameter spaces using probabilistic surrogate models [21].

### 2.7. Class Imbalance Mitigation

Class imbalance, a common challenge in biomedical datasets, was addressed using the Synthetic Minority Oversampling Technique (SMOTE) with k = 5 nearest neighbors [22]. SMOTE synthetically generated minority class samples by interpolating feature vectors between existing instances, achieving a balanced distribution. This approach reduced overfitting risks and provided robust estimates of performance metrics.

### 2.8. SHAP Analysis for Interpretability

To ensure clinical relevance and transparency, SHAP values were computed using the TreeExplainer algorithm from the SHAP library (v0.42.1). SHAP values, rooted in cooperative game theory, quantify the contribution of each feature to individual predictions. Global feature importance was derived by averaging the absolute SHAP values across the dataset, identifying biomarkers with the strongest influence on the model outcomes. For local interpretability, summary plots visualized feature effects on specific predictions, enabling clinicians to validate biologically plausible relationships. The use of TreeExplainer, optimized for tree-based models like LightGBM, ensured computational efficiency and exact SHAP value calculations [23].

## 3. Results

The original dataset included metabolomics data obtained from serum samples of 103 breast cancer patients and 31 healthy controls. To address the original dataset’s class imbalance, SMOTE was applied prior to model training to synthetically generate minority class samples. This ensured balanced class distributions for reliable model development and prevented bias toward majority class predictions. Since class imbalance can lead to biased results in machine learning-based prediction models, the SMOTE method was used in this study. After SMOTE, the class distribution was balanced, and further analyses were performed on the balanced data. After eliminating class imbalance, we evaluated the effects of different feature selection strategies on the classification performance to explain the structure in the data with fewer metabolites. The MOFS approach achieved the best performance of all metrics: accuracy (99.59%), F1-score (99.74%), AUC (99.39%). After the MOFS approach, the Boruta method also achieved particularly high sensitivity (99.91%) and specificity (98.18%). The MI and sPLS methods achieved relatively lower specificity and poorer performance in terms of classification results. The current findings show that the MOFS approach, which is based on combining features selected by more than one method, can be more stable in the detection of breast cancer, which is a binary classification problem. Therefore, metabolomic data selected by the MOFS approach were used in further analyses (Table 1).

Table 2 shows the performance metrics for breast cancer prediction using the LightGBM, AdaBoost, and Random Forest algorithms, which are tree-based machine learning approaches. All models were evaluated with comprehensive performance metrics. In terms of accuracy, the LightGBM model showed the highest performance with 86.6% (95% CI: 81.9–91.3). Its F1-Score was higher than that of the other models, with 87.0% (95% CI: 82.3–91.6), and especially in terms of sensitivity, it had the best performance, with 89.1% (95% CI: 81.3–94.4). This is an important result because one of the main objectives of the study was to correctly distinguish the positive class, i.e., breast cancer patients, and the sensitivity metric was evaluated as an important performance result. Therefore, the highest sensitivity value obtained by the LightGBM model showed that it was stronger than the other models in terms of its capacity to correctly recognize positive class samples. These results reveal that the LightGBM model can be used effectively, especially in applications where minimizing false negatives is important. In addition, the specificity of the LightGBM model was calculated to be 84.2% (95% CI: 75.6–90.7), and the AUC was 91.6% (95% CI: 86.6–96.5). Meanwhile the AdaBoost model came in second with 83.7% accuracy (95% CI: 78.6–88.8) and an 83.9% F1-Score (95% CI: 78.8–89.0); in addition, its sensitivity was calculated to be 85.1% (95% CI: 76.7–91.4), and its specificity was calculated to be 82.2% (95% CI: 73.3–89.1). The Random Forest model showed lower performance than the other two models: accuracy, 80.2% (95% CI: 74.7–85.7); F1-Score, 80.4% (95% CI: 74.9–85.9); sensitivity, 81.2% (95% CI: 72.2–88.3); specificity, 79.2% (95% CI: 70.0–86.6). While LightGBM demonstrated superior performance, it is worth noting that the confidence intervals overlap considerably among models, particularly between LightGBM and AdaBoost (Table 2).

This overlap suggests that the performance differences, while consistent, may not be statistically significant in all evaluation dimensions. The broadest confidence intervals were observed in the sensitivity metrics, indicating greater variability in the models’ ability to correctly identify positive cases. Overall, these results suggest that LightGBM offers the most robust performance for this classification task, with particular strength in identifying positive cases, though AdaBoost provides competitive performance that may be suitable in scenarios where computational efficiency is prioritized in this process.

**Figure 1:**
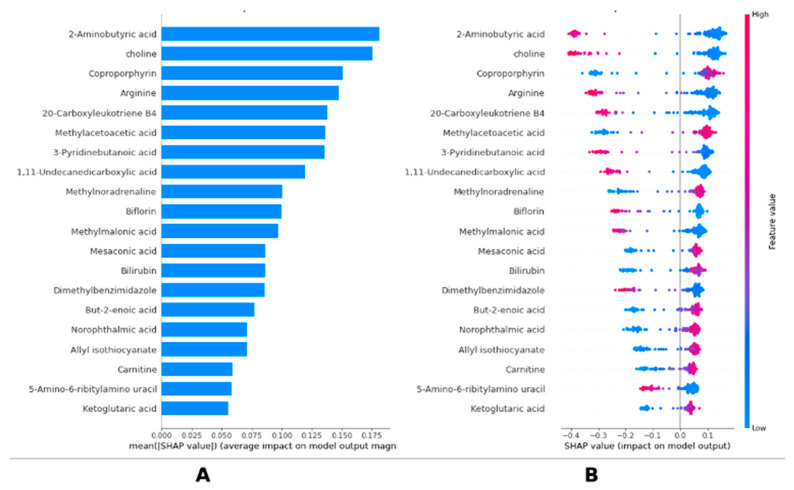
SHAP-based interpretation of LightGBM predictions in breast cancer. (**A**) Global feature importance of metabolites based on mean absolute SHAP values. Bar plot is shown displaying mean absolute SHAP values for each metabolite included in final LightGBM model. Higher SHAP values indicate greater influence on model’s classification outcome. (**B**) Individual SHAP value distributions across samples highlighting metabolite-level effects. Bee-swarm plot illustrates individual SHAP values for each sample and metabolite. Dot colors indicate metabolite concentration (pink = high value; blue = low value), revealing how variations in metabolite levels affect model predictions.

Figure 1 shows the importance ranking of metabolomics based on the SHAP explainability method of the LightGBM model and the effects of metabolomics on the model output. Figure 1A, the ranking of metabolomics according to average SHAP values, shows that 2-Aminobutyric acid is the most effective metabolite in the model, followed by choline, coproporphyrin, arginine, and 20-Carboxyleuthoriene B4, respectively. Figure 1B visualizes the SHAP values of individual data points and the effects of low (blue) and high (pink) values of metabolites on the model output. In particular, it was determined that low values of 2-Aminobutyric acid, choline, arginine, and 20-Carboxyleuthoriene B4 metabolites, in addition to high levels of coproporphyrin metabolites, increased the risk of breast cancer. These results suggest that biochemical compounds play an important role in the decision-making process of the LightGBM model and that these compounds are determinant in predicting clinical outcomes. The metabolites identified in this study, including 2-Aminobutyric acid, choline, and coproporphyrin, are established to be critical regulatory molecules involved in amino acid and lipid metabolism, which are often dysregulated in breast cancer. They may signal underlying tumor biology and may help uncover or predict disease progression (Figure 1). Detailed group-wise descriptive statistics (mean ± SD) and significance levels (*p* < 0.001) for the 20 key metabolites identified through SHAP analysis are reported in Supplementary Table S1.

## 4. Discussion

With the current study, we provide the necessary information to determine the implications of using machine learning models in breast cancer prediction using metabolomics data. With the SMOTE method, the class imbalance problem was solved, and an adequately sized balanced dataset was obtained for reliable and unbiased predictions. This was an important step, as imbalanced datasets can be biased towards obtaining biased outcomes, especially when predicting for minority class instances [22]. Moreover, the scientific study [24] rightly points out that oversampling techniques like SMOTE improve not just model accuracy but also fairness in model prediction in healthcare data science. The emerging literature supporting the utility of metabolomics data as a source of biosignatures pointing to a disease state is summarized by the suggestion that such data might be leveraged to facilitate early diagnosis and personalized interventions [25].

In this study, different feature selection approaches were applied to distinguish breast cancer patients from healthy controls. Using high-dimensional serum metabolome data, tree-based machine learning prediction models were developed, the optimal breast cancer prediction model was determined by comparing the results with comprehensive performance metrics, and clinical explanations of the optimal prediction model were obtained with SHAP and an XAI method. The results revealed that these methods play a critical role in both model success and clinical applicability. The feature selection methods used included various statistical, regression-based, and ensemble-based algorithms such as MI, sPLS, Boruta, and MOFS, and through these approaches, individual feature effects, interactions between features, and the level of generalizability of the model were evaluated in a multifaceted manner. The MOFS strategy, which is a combination of these methods, showed superior success in both classification metrics and allowed for the acquisition of a biologically meaningful and reproducible feature subset, as it possessed features common to more than one algorithm. The models obtained with MOFS showed superior performance in correctly distinguishing both positive and negative classes, especially in terms of AUC and F1-score criteria, leaving other methods behind. When evaluated from a clinical perspective, it is seen that such feature selection strategies not only provide statistical significance, but also have the potential to increase the accuracy of diagnostic processes by reducing false positive and false negative rates and to prevent unnecessary interventions. This strengthens the reliability and effectiveness of model-based diagnostic support systems, especially in cases such as breast cancer, where early diagnosis and correct classification directly affect the course of the disease.

The performance of our three tree-based models, as measured based on accuracy, F1-score, sensitivity, and specificity, clearly indicates that the LightGBM model outperforms the other two models evaluated. Notably, LightGBM achieves a sensitivity rate of 89.1%. This is good because the model succeeded in not predicting false negatives, which would prevent the prompt diagnosis and treatment of breast cancer. Indeed, the model performs better than AdaBoost and Random Forest in other research that looks at how well it matches complex biomedical data with high-dimensional features [20]. LightGBM has demonstrated exceptional accuracy in breast cancer prediction, achieving an average accuracy of 99.12% with high specificity and precision [26]. While LightGBM achieved the highest accuracy (86.6%, 95% CI: 81.9–91.3), overlapping confidence intervals with AdaBoost (83.7%, 95% CI: 78.6–88.8) suggest that performance differences may not reach statistical significance in all metrics. However, LightGBM’s superior sensitivity (89.1% vs. AdaBoost’s 85.1%) remains clinically meaningful for early BC detection, where minimizing false negatives is critical. Cancer prediction using multi-omics data using LightGBM has emerged as an algorithm of choice as it offers scalability and robustness across different studies. Because it can process large datasets and complex omics interactions, it is well suited to increasing diagnostic accuracy and patient outcomes. This section presents some highlights of LightGBM’s performance in this respect [27]. However, previous studies showed that Random Forest is a robust algorithm in most cases but has been observed to lack the nuanced adaptability of boosting algorithms, notably for datasets that require feature interactions [28]. Such comparative analyses reaffirm LightGBM’s potential as a robust tool for breast cancer prediction in metabolomics research, particularly when paired with advanced feature selection and explainability techniques like SHAP. Previous studies in breast cancer research have shown the dysregulation of amino acid and lipid metabolism, as reflected by changes in 2-Aminobutyric acid, choline, coproporphyrin. In addition to their diagnostic utility, these metabolites may also be used as potential therapeutic targets [29].

The specificity of the LightGBM model, which reaches 84.2%, also means that it reduces false positives to a minimum; that is, healthy people are much less likely to be classified as having breast cancer. While LightGBM’s 84.2% specificity minimizes false positives, its 89.1% sensitivity surpasses CA 15-3′s AUC of 0.610–0.684, highlighting its potential for early BC detection. This balance aligns with clinical needs, where high sensitivity is prioritized for screening, but specificity remains critical to avoid overtreatment. In clinical decision-making, this balance between sensitivity and specificity is crucial, as in the tool, it both supports early detection and eliminates needless treatment [30]. In comparative studies coupled with big datasets, the LightGBM model often favors balanced metrics in optimizing these tradeoffs, which are of particular importance in healthcare [31]. In addition, the model shows high positive (84.9%) and negative (88.5%) predictive values, and can therefore be used for clinical and diagnostic purposes because of its high ability to correctly identify both true positive and true negative cases. The inclusion of LightGBM in diagnostic workflows has been supported by its performance in a range of medical datasets with high specificity and predictive values. The capability of the model to be used on complex data and its ability to make accurate predictions make it an important tool in healthcare settings [32]. Recently, relevant studies have investigated how machine learning algorithms, such as LightGBM, can be used to analyze complex metabolomic data for cancer diagnostics. It has been demonstrated that the combination of metabolomics with other omics data improves diagnostic accuracy and provides a more in-depth insight into the biology of cancer [33].

Another important aspect of this study is explainability. We then selected key metabolites that materially affected the predictions of our LightGBM model using the SHAP method. Their potential as biomarkers for breast cancer is indicated by the ranking of metabolites, ranked from highest (2-Aminobutyric acid, choline, coproporphyrin) to lowest (fumarate, alanine, itaconate). Low 2-Aminobutyric acid, choline, and arginine and high coproporphyrin could be related to elevated breast cancer risk. These results are consistent with previous research showing that BC risk and progression are associated with changes in amino acid metabolism, including arginine, choline, and related metabolites, which may serve as targets for diagnosis and therapy [29]. Furthermore, recent studies are confirming the important role of these metabolites in cancer diagnostics [34]. For instance, amino acid metabolism changes are important indicators of tumorigenesis and are useful for predicting prognoses, predicting immune responses, and predicting the treatment outcomes of many cancers, thus supporting their use in predictive models. In addition, the combination of SHAP explainability methods guarantees that such biological insights are preserved and accessible for clinical interpretation, connecting the computational predictions to biochemical understanding. To assess specificity, we compared metabolite levels in BC patients with those ingastric/colorectal cancer cohorts. Carnitine (193.845 ± 43.776 in BC vs. 86.098 ± 20.668 in controls) showed BC-specific elevation, whereas 3-Pyridinebutanoic acid (63.701 ± 12.346 in BC vs. 149.996 ± 25.558 in controls) was uniquely reduced in BC. These findings suggest that the identified metabolites may serve as BC-specific biomarkers rather than general cancer indicators. Taken collectively, these results suggest that metabolite-driven insights can inform both early detection strategies and targeted therapeutic interventions, and ultimately increase the utility of predictive models in oncology. Beyond the biological effects of the metabolites identified in this study (e.g., choline, 2-aminobutyric acid), examining molecular pathways associated with aggressive phenotypes of breast cancer may add depth to our findings. In particular, transcription factors such as Hippo signaling pathway effectors YAP (Yes-related protein), TAZ (transcriptional coactivator with PDZ-binding motif), and TEAD (TEA domain family) show close associations with the epithelial–mesenchymal transition (EMT) master regulators ZEB, Snail, and Twist. EMT master regulators such as ZEB and Snail, along with the Hippo pathway effectors YAP, TAZ, and TEAD, are increasingly recognized for their roles in driving aggressive phenotypes in breast tumors. These factors interact to promote tumor progression, invasion, metastasis, and therapy resistance, particularly in aggressive breast cancer subtypes [35,36,37,38]. High nuclear expression of YAP, TAZ, and TEAD is significantly correlated with increased levels of ZEB and Snail in breast phyllodes tumors, especially in higher-grade, more aggressive cases [36]. ZEB1 forms complexes with YAP and AP-1 (FOSL1/JUN), activating tumor-promoting genes and reinforcing EMT, particularly in aggressive claudin-low breast cancer subtypes [37]. The interaction between YAP/TAZ-TEAD and EMT regulators amplifies malignant features, including enhanced proliferation, invasion, and metastatic potential [36,37,39]. In the literature, such molecular interactions emphasize the integration of metabolic disorders as well as signaling pathways in the early diagnosis of breast cancer. In this context, future studies may provide a more holistic perspective on breast cancer biology by correlating identified metabolites with Hippo-EMT interactions. How metabolic changes such as choline deficiency, identified as important biomarker candidates in the present study, affect YAP/TAZ activation or the EMT process can be investigated experimentally in future studies. Such multidisciplinary approaches may contribute to the development of both diagnostic models and treatment strategies.

In addition to the interpretability of the LightGBM model, SHAP-based explainability offers visual insights into the impact that metabolite levels have on prediction at the population and individual levels. SHAP analysis bridges computational predictions and clinical practice. For example, low 2-Aminobutyric acid and choline levels (pink in Figure 1B) could guide clinicians to prioritize imaging or biopsies in high-risk patients, while high coproporphyrin may signal metabolic vulnerabilities for targeted therapy. Fostering trust amongst clinicians and researchers in AI-driven decision support systems is critical. For clinical adoption, important studies [28] have recommended that model transparency is key because it allows practitioners to understand why a prediction was made [40]. These explainability findings also provide guidance for further experimental studies to verify the biological roles of the identified metabolites in breast cancer pathogenesis. For instance, a research paper points out that SHAP analysis enhances transparency and trustworthiness in supervised machine learning models for drug development, improving their impact on clinical decisions [41]. SHAP-based explainable machine learning identifies patient-specific biomarker genes that can be used to formulate an effective therapy for lung cancer patients or to aid in early detection and therapeutic targets [42]. Collectively these findings will drive the development of more comprehensive and patient-specific diagnostic models for oncology with computational outputs producing biologically relevant and clinically actionable information. Beyond the machine learning-based metabolomics approaches presented in this study, AI and image processing technologies have also made significant progress in cancer diagnosis [43]. Especially in malignancies where image-based diagnosis methods are critical, such as liver and colorectal cancer, deep learning algorithms have accelerated and increased the accuracy of histopathological and radiological image analyses [44,45,46]. For example, AI models trained to classify lesions in computed tomography (CT) or magnetic resonance (MR) images in liver cancer diagnosis have shown higher sensitivity and specificity than experts. Similarly, algorithms developed for the automatic determination of epithelial-cell structure and tumor boundaries in the histopathological evaluation of colorectal cancer tissue samples have reduced the workload of pathologists and increased early diagnosis rates. These methods can extract a greater number of features (e.g., tissue texture, cell morphology) compared to traditional analyses and can detect microscopic changes at a level that the human eye cannot detect. In addition, real-time AI systems developed for polyp detection during endoscopic imaging help minimize false-negative results during colonoscopy. In the future, the integration of such image-based AI approaches with metabolomics data may contribute to the development of personalized treatment strategies by providing a more holistic perspective on cancer biology [47,48,49].

This study highlights some clinical implications and demonstrates the progress in developing a method for the early diagnosis and management of breast cancer utilizing the LightGBM tool. The model aligns with recent trends in precision medicine, where information about biomarker profiles assists in early intervention guided towards individualized profiles [50]. At the same time, the integration of machine learning with SHAP explainability addresses the severe lack of conventional diagnostic methods and enables the transparency of model predictions that are readily interpreted by clinicians. As reported by study [51], the integration of AI-driven analytics, e.g., machine learning, with metabolomics has the potential to dramatically reduce diagnostic timelines—by enabling faster disease classification, biomarker identification, and early diagnosis for conditions including cancers. The relevance of metabolites (e.g., 2-aminobutyric acid, choline, coprophyrin) highlighted by the SHAP analysis to breast cancer biology may be further supported by recent studies. For example, the antioxidant effects of 2-aminobutyric acid may cause the apoptosis of tumor cells by reducing oxidative stress via glutathione synthesis, which, in turn, inhibits breast cancer growth. Choline deficiency causes impairments in phospholipid synthesis and the loss of cell membrane stability, which has been shown to promote metastasis by triggering the EMT process. Similarly, porphyrin derivatives such as coprophyrin have been associated with oxidative stress and have been suggested to promote malignancy by increasing ROS production in breast cancer cells. In a broader context, the effects of arginine and non-essential amino acids on breast cancer progression are also noteworthy. It has been shown that their deficiency can limit tumor cell proliferation and angiogenesis. Furthermore, increased carnitine levels provide energy by increasing lipid β-oxidation, and this metabolite plays a critical role in the invasion of breast cancer cells. The pathophysiological roles of these metabolites support the biological consistency of the prediction made by the SHAP analysis [52,53,54,55,56,57].

Despite these promising results, some limitations of this study warrant consideration. First, the relatively small sample size may limit the generalizability of the findings. Future studies should aim to validate the model on larger, independent cohorts. Second, while SMOTE effectively addressed class imbalance, it may not fully capture the complexity of real-world data distributions. Advanced oversampling techniques, such as SMOTE combined with Tomek links, could be explored to further enhance data quality. High classification metrics, such as the 99.59% accuracy and 99.91% sensitivity obtained with the MOFS method, were obtained thanks to the large effect size in the dataset (Cohen’s d = 4.14) and class balance with SMOTE. However, for more reliable generalization, external validation is recommended on independent cohorts (e.g., data collected from different centers). To increase the predictive power of these models, integration with other omics data (e.g., transcriptomics and proteomics) could further improve the predictive power of these models and provide a more complete understanding of breast cancer biology, leading to the creation of personalized treatment strategies. Further research needs to be performed to integrate multi-omics data comprising transcriptomics and proteomics, in order to build more elaborate diagnostic models that reflect the intricacies of breast cancer biology. The identified biomarkers need to be validated in prospective clinical trials in the real world, to determine whether they can be utilized to guide early intervention and personalized treatment strategies.

Finally, the LightGBM model’s metabolomics data for breast cancer prediction, when combined with SHAP explainability, provides a powerful and interpretable path. The identification of key metabolites is a prerequisite for conducting further studies into potential biomarkers and therapeutic targets. The results of this study provide insight into the utility of combining machine learning and metabolomics to move personalized medicine in oncology forward. A reported study [33] demonstrates that the integration of transcriptomics, proteomics, and metabolomics data is a potent method for incorporating additional information and gaining a more comprehensive understanding of a biological system. This extends beyond the basic patterns and relationships that a single omics approach could reveal, revealing intricate networks and relationships among various organizational levels. Using these combined methods shows that LightGBM’s machine learning models could help with data-driven, personalized care based on patient data in oncology and other fields as well.

## 5. Conclusions

The present study demonstrates the potential of combining advanced serum-based metabolomics profiling with interpretable machine learning (LightGBM-SHAP) to improve breast cancer diagnosis. By applying a robust combination of the MOFS and XAI methods, we identified a panel of serum metabolites that effectively discriminate breast cancer patients from healthy controls with high predictive performance. More importantly, the biological significance of key metabolites such as 2-Aminobutyric acid, choline, coproporphyrin, arginine, and carnitine is also supported by recent studies linking these markers to critical cancer-related pathways such as oxidative stress, lipid metabolism, and epithelial–mesenchymal transition. The incorporation of SHAP analysis into the methodology increased transparency and clinical explainability by interpreting the decision-making process of the model. Our findings suggest that combining metabolomics with interpretable machine learning may be useful for developing reliable, biologically informed diagnostic tools for breast cancer and potentially other malignancies.

## Figures and Tables

**Table 1:** Comparative results of model performance of different feature selection approaches in breast cancer detection.

Methods	Accuracy	Sensitivity	Specificity	F1-Score	AUC
MI	0.993240	0.997359	0.978788	0.995667	0.990043
sPLS	0.992567	0.998225	0.972727	0.995265	0.986991
MOFS	0.995934	0.999091	0.984848	0.997399	0.993939
Boruta	0.995260	0.999091	0.981818	0.996976	0.991970

**Table 2:** Performance metrics for breast cancer prediction using LightGBM, AdaBoost, and Random Forest algorithms.

Metric/Model	LightGBM	AdaBoost	Random Forest
Accuracy	0.866 (0.819–0.913)	0.837 (0.786–0.888)	0.802 (0.747–0.857)
F1-Score	0.87 (0.823–0.916)	0.839 (0.788–0.89)	0.804 (0.749–0.859)
Sensitivity	0.891 (0.813–0.944)	0.851 (0.767–0.914)	0.812 (0.722–0.883)
Specificity	0.842 (0.756–0.907)	0.822 (0.733–0.891)	0.792 (0.7–0.866)
AUC	0.916 (0.866–0.965)	0.891 (0.836–0.946)	0.861 (0.802–0.921)

## Data Availability

The raw data supporting the conclusions of this article will be made available by the authors on request.

## Supplementary Materials

The following supporting information can be downloaded at: https://www.mdpi.com/article/10.3390/medicina61061112/s1, Table S1: Descriptive statistics of the significantly expressed metabolites found in breast cancer.
