# Supplementary material for: Interpretable Machine Learning for Serum-Based Metabolomics in Breast Cancer Diagnostics: Insights from Multi-Objective Feature Selection-Driven LightGBM-SHAP Models
Source: Medicina (Kaunas). 2025 Jun 19;61(6):1112. doi: 10.3390/medicina61061112 (PMC12195222; doi:10.3390/medicina61061112)
Supplement: Supplementary file 1 [file medicina-61-01112-s001.zip › medicina-3659916-supplementary.pdf]

Table S1. Descriptive statistics of the significantly expressed metabolites regarding breast cancer

Metabolite	Control	Breast Cancer	*P*-value
	Mean ± SD	Mean ±SD	
2-Aminobutyricacid	8.278 ± 2.424	0.914 ± 0.668	<0.001*
Choline	23.608 ± 3.940	3.871 ± 0.886	<0.001*
Coproporphyrin	0.153 ± 0.081	5.381 ± 2.764	<0.001*
Arginine	4.651 ± 1.319	1.263 ± 0.333	<0.001*
20-CarboxyleukotrieneB4	1.507 ± 0.649	0.083 ± 0.100	<0.001*
Methylacetoaceticacid	2.836 ± 3.319	30.206 ± 7.100	<0.001*
3-Pyridinebutanoicacid	149.996 ± 25.558	63.701 ± 12.346	<0.001**
1.11-Undecanedicarboxylicacid	1.426 ± 2.073	0.024 ± 0.029	<0.001*
Methylnoradrenaline	1.118 ± 0.684	5.234 ± 1.589	<0.001*
Biflorin	1.633 ± 0.816	0.007 ± 0.062	<0.001*
Methylmalonicacid	13.082 ± 10.178	2.143 ± 0.585	<0.001*
Mesaconicacid	2.777 ± 1.553	43.140 ± 15.793	<0.001*
Bilirubin	0.236 ± 0.630	7.244 ± 4.467	<0.001*
Dimethylbenzimidazole	3.928 ± 2.899	0.977 ± 0.326	<0.001*
But-2-enoicacid	0.334 ± 0.381	3.354 ± 1.197	<0.001*
Norophthalmicacid	0.655 ± 0.595	4.699 ± 2.141	<0.001*
Allylisothiocyanate	0.765 ± 0.443	4.778 ± 1.751	<0.001*
Carnitine	86.098 ± 20.668	193.845 ± 43.776	<0.001**
5-Amino-6-ribitylaminouracil	0.839 ± 0.273	0.141 ± 0.106	<0.001*
Ketoglutaricacid	0.723 ± 2.954	4.190 ± 1.478	<0.001*

*: Mann-Whitney U test, **: Independent Samples *t* test
